# Impact of hemoglobin levels on acute ischemic stroke severity

**DOI:** 10.3389/fneur.2025.1534746

**Published:** 2025-04-28

**Authors:** Shaima Abuhulayqah, Fajar Abdulrazzak Aldulijan, Alaa Nabil Turkistani, Albatoul Fahad Almulhim, Cereen Fahad Almulhim, Shahid Bashir, Eman Nassim Ali

**Affiliations:** ^1^Department of Adult Neurology, King Fahad Hofuf Hospital, Hofuf, Saudi Arabia; ^2^Department of Family Medicine, Johns Hopkins Aramco Healthcare, Dhahran, Saudi Arabia; ^3^Neurosciences, King Faisal Specialist Hospital and Research Centre, Jeddah, Saudi Arabia; ^4^Department Neurology, King Fahad Medical City, Riyadh, Saudi Arabia; ^5^Department of Pathology and Laboratory Medicine, King Fahad Specialist Hospital, Dammam, Saudi Arabia; ^6^Neuroscience Center, King Fahad Specialist Hospital, Dammam, Saudi Arabia; ^7^King Salman Center for Disability Research, Riyadh, Saudi Arabia; ^8^Department of Adult Neurology, King Fahad Specialist Hospital, Dammam, Saudi Arabia

**Keywords:** hemoglobin levels, acute ischemic stroke, stroke severity, anemia and stroke, stroke risk factors, stroke assessment

## Abstract

**Introduction:**

Stroke is one of the most common causes of disability and mortality worldwide. In Saudi Arabia, it is a crucial health issue. Ischemic stroke is the most common type of stroke in this area, and understanding its relationship with hemoglobin (Hgb) levels is vital. To date, no study has established an exact relationship between Hgb levels and stroke severity. This study assessed the association between Hgb levels and the severity of acute ischemic stroke (AIS) at presentation.

**Methods:**

We conducted a retrospective study of patients admitted and diagnosed with AIS between 2013 and 2017. The exclusion criteria included other stroke types (such as hemorrhagic or venous infarction), patients with a history of internal bleeding, and pregnant and lactating women. The patients were divided into three groups based on Hgb levels: low, average, and high. Correlations were analyzed between these groups and the National Institutes of Health Stroke Scale (NIHSS) scores, stroke outcomes at discharge (cured, improved, or mortality decreased), and stroke subtype, as determined and classified by the TOAST classification criteria.

**Results:**

The Pearson correlation coefficient showed a weak positive correlation between Hgb levels and NIHSS scores. Neither stroke outcomes nor stroke types showed significant correlations with mean Hgb level.

**Conclusion:**

The results of this retrospective study on a small cohort of patients diagnosed with AIS indicate that higher Hgb levels at hospital admission are associated with greater stroke severity, as measured by the NIHSS score. However, no significant effect was observed on stroke outcome at discharge or the TOAST classification.

## Introduction

Stroke is one of the leading causes of disability and mortality worldwide. According to the World Health Organization (WHO), approximately 15 million people worldwide experience a stroke each year, with 5.5 million deaths and another 5 million living with enduring disabilities (1). Previous studies have described a U-shaped relationship between hemoglobin (Hgb) levels and short-term stroke mortality (2–4). Patients with low Hgb levels had higher mortality rates at all-time points compared to those with healthy Hgb levels. At the same time, high Hgb levels were also associated with elevated mortality rates. However, these studies did not account for different stroke types, which may have acted as confounding factors (5, 6).

Studies have shown that patients with anemia on admission have a higher risk of mortality within the first 3 years after stroke than those with healthy Hgb levels (3, 7, 8). Another study showed that among ischemic stroke survivors, a high Hgb level on admission was associated with more severe stroke, more significant disability at discharge, and higher 30-day mortality; however, a low Hgb level was not significantly associated with neurological impairment but was associated with higher 90-day mortality following a more extended period of stay in an acute stroke care facility (9).

Hgb is the essential structure for oxygen transport in red blood cells (RBCs) and helps maintain a stationary balance between oxygen supply and demand in all body tissues. Because a mismatch between oxygen supply and demand is the mainstay of the pathophysiology of acute ischemic stroke (AIS), identifying the factors contributing to this imbalance is essential. The pathophysiological pathways through which low and high Hgb levels affect stroke presentation and outcomes remain unclear (2–4, 10). Some investigators have hypothesized that low Hgb levels may induce hypoxia, while high Hgb levels may increase blood viscosity, leading to diminished cerebral blood flow (4).

In Saudi Arabia, stroke has become one of the most significant health concerns (11, 12). Although the incidence and prevalence of stroke in KSA are lower compared to other countries in the region (13), risk factors such as systemic hypertension (38%), diabetes mellitus (37%), and heart disease are highly prevalent (14, 15).

Because ischemic stroke is the most common type of stroke in this region, understanding the relationship between Hgb levels and this type of stroke is essential (13). Therefore, this study aimed to determine the relationship between Hgb levels and the severity of acute ischemic stroke at presentation.

## Materials and methods

This single-center retrospective chart review included all patients diagnosed and treated with acute ischemic stroke (AIS) between 2013 and 2017. We collected data from electronic and paper-based medical records. The study protocol was reviewed and approved by the Research Ethics Board of King Fahad Specialist Hospital, Dammam, and complied with the principles outlined in the Declaration of Helsinki. The inclusion criteria included a diagnosis of AIS, which is defined as an acute neurologic deficit lasting >24 h and caused by a cerebrovascular etiology (atherosclerotic or embolic) and occurring within the acute to subacute stage (up to 1 week from onset) (16), age ≥ 18 years, confirmation of diagnosis by MRI, and documented Hgb level at initial presentation. Exclusion criteria were patients with other types of stroke, a history of internal bleeding, and pregnant or lactating women.

Hgb levels were measured using the initial blood samples drawn upon arrival at the emergency department within the first 4 h of AIS onset. Hgb levels were classified as low (<12 g/dL in women, <13 g/dL in men), normal, and elevated (>15.5 g/dL in women and > 17.0 g/dL in men), based on the WHO criteria. The primary outcome was the correlation between Hgb levels and AIS severity at admission, which was determined by the National Institutes of Health Stroke Scale (NIHSS) score. Secondary outcomes included the effects of Hgb levels on discharge status (cured, improved, or deceased), stroke type (cortical, subcortical, cortical/subcortical, infratentorial), and stroke classification according to the TOAST classification system. This system, developed for the Trial of Org 10,172 in Acute Stroke Treatment (TOAST), categorizes ischemic stroke subtypes primarily based on etiology (17).

We performed statistical analysis using SPSS software, and Pearson correlation was used to test the primary outcome. For the secondary outcomes, the groups were compared using the chi-square test for categorical variables and the Analysis of Variance (ANOVA) for continuous variables. Multiple variables (diabetes, coronary artery disease, atrial fibrillation, hypertension, dyslipidemia, and history of stroke) were included in a linear regression model to assess its effect on the NIHSS scores.

## Results

A total of 44 subjects [16 (36%) women and 28 men (64%)], with a mean age of 68.8 years (standard deviation [SD] = 13.5, 95% confidence interval [CI]: 64.5–73.1, range 37–92 years), were included in the study, Mean NIHSS scores across the sample were 4.9 (SD = 2.3, range 1–10), and Hgb levels averaged 12.8 g/dL (SD = 2.0, 95% CI: 12.0–13.6, range: 8.8–17.3). Furthermore, six patients had cortical stroke, eight had cortical/subcortical stroke, eight had infra-tentorial stroke, and 22 had subcortical stroke. Demographics are summarized in Table 1.

**Table 1:** Demographics.

Variables	*N*	Mean (SD)
Gender		
Male (n)	16	
Female (n)	28	
NIHSS		
Mean (SD)		4.9 (2.3)
Hgb level		
Mean (SD) g/dL		12.8 (2.0)
Age		
Mean (SD) years		68.8 (13.5)
Discharge status		
Cured	3	
Improved	39	
Transferred	1	
Mortality	1	
Type of stroke		
Subcortical	22	
Cortical/subcortical	7	
Brainstem	6	
Cortical	6	
Cerebeller	2	
TOAST classification		
Small vessel occlusion	21	
Atherosclerotic	15	
Embolic	6	
Stroke of undetermined etiology	1	
Stroke of other determined etiology	1	

**Figure 1:**
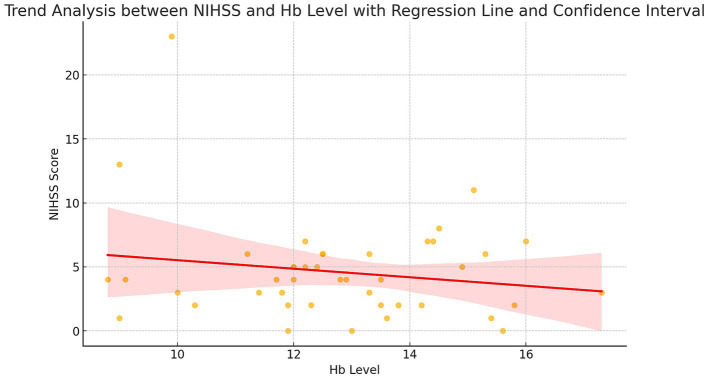
A weak negative correlation is observed between Hgb levels and NIHSS scores, with a correlation coefficient of *r* = −0.172 and a significance level of 0.270.

The primary outcome, correlation analysis between NIHSS and Hgb levels, indicated a weak negative relationship (*r* = −0.17, 95% CI for r: −0.46, 0.15, *p* = 0.27) (Figure 1).

For the secondary outcomes, the mean Hgb levels across different discharge statuses are shown in Table 2. Three patients achieved a cure (mean Hgb 13.6 g/dL), 39 showed improvement (mean Hgb 12.7 g/dL), and one patient died (mean Hgb 13.2 g/dL). No significant differences in mean Hgb levels were observed among these groups.

**Table 2:** Mean hemoglobin level and different discharge status.

Discharge status	n	Mean Hgb level (g/dL)
Cured	3	13.6
Improved	39	12.7
Mortality	1	13.2

The NIHSS scores were analyzed by stratifying the patients into age groups (30–49, 50–59, 60–69, 70–79, and > 80 years). The median NIHSS scores were relatively stable across the age groups, with no significant differences observed (*p* = 0.48). For example, the 60–69 age group had a median NIHSS score of 4.7 (95% CI: 3.9–5.6), while the 70–79 group had a median score of 5.1 (95% CI: 4.2–6.1).

The mean Hgb levels for the different TOAST classes of ischemic stroke are shown in Table 3, with the highest levels observed in strokes of atherosclerotic origin, followed by embolic strokes, small vessel occlusion, and the lowest in strokes due to other etiologies (*p* = 0.007).

**Table 3:** Effect of hemoglobin level on stroke TOAST classification.

TOAST classification	n	Mean Hgb level (g/dL)	SD	Median Hgb level (g/dL)
Atherosclerotic	15	13.6	1.7	13.5
Embolic	6	13.2	2.5	12.6
Small-vessel occlusion	21	12.4	1.7	12.0
Stroke of other etiology	2	8.9	0.1	8.9
Total	44	12.8	2.0	12.7

Regarding the effect of various comorbidities on the severity of AIS, only the presence of atrial fibrillation showed a significant effect on the NIHSS score (*p* < 0.05) (Table 4).

**Table 4:** Linear regression model assessing confounder effects on the NIHSS score.

Predictor	Unstandardized coefficients (B)	Std. error	Standardized coefficient (beta)	t	Sig. (*p*-value)
Constant	1.860	1.335	-	1.393	0.172
CAD	−0.225	1.840	−0.021	−0.122	0.903
AF	4.802	1.706	0.425	2.815	0.008
DM	0.560	1.372	0.068	0.408	0.686
HTN	1.136	1.329	0.136	0.855	0.398
Dyslipidemia	1.520	1.426	0.194	1.066	0.293
Stroke	1.024	1.464	0.110	0.700	0.489

**Figure 2:**
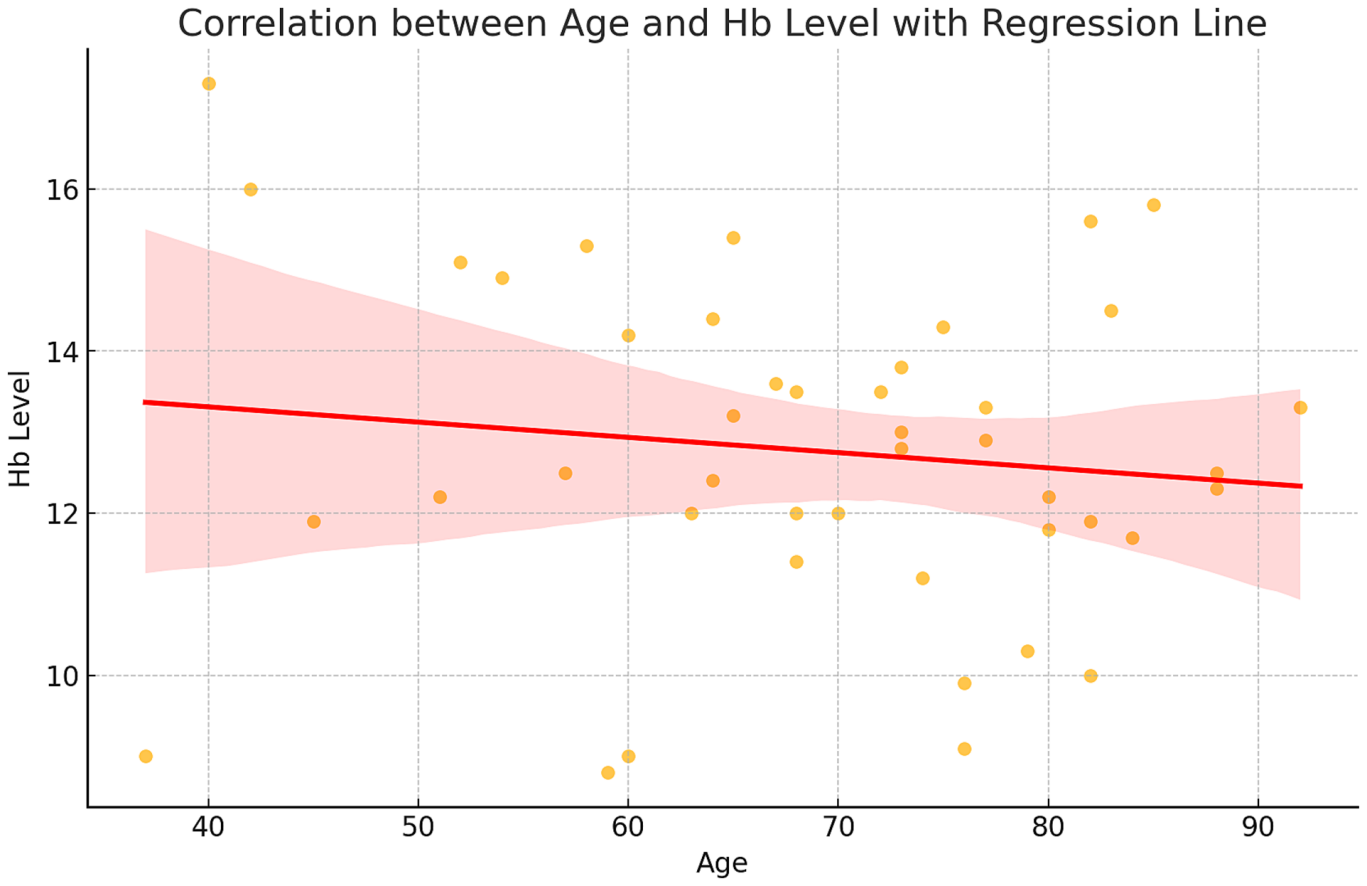
Correlation between age and Hgb level with regression line.

The relationship between age and Hgb levels was evaluated, revealing a weak negative correlation (*r* = −0.13, 95% CI for r: −0.42 to 0.18, *p* = 0.39). Hgb levels slightly declined with age, although this trend was not statistically significant. The mean Hgb level in the sample was 12.8 g/dL (SD = 2.0, 95% CI: 12.0–13.6), with a range from 8.8 to 17.3 g/dL (Figure 2).

Comparing NIHSS scores, Hgb levels, and age across stroke types showed significant differences among the categories. Cortical/subcortical, Brainstem and cerebellar strokes had higher mean NIHSS scores and slightly lower mean Hgb levels (brainstem: 12.3 g/dL, 95% CI: 11.4–13.2) compared to cortical strokes, which had lower NIHSS scores and marginally higher Hgb levels. Age also varied by stroke type, with patients having cortical strokes generally being older (mean age: 77.3 years, 95% CI: 72.4–82.2) compared to those with cerebellar or subcortical strokes.

The mean Hgb levels did not differ significantly across these types (*p* = 0.376) (Table 5).

**Table 5:** Stroke type NIHSS, Hgb level and age.

Stroke type	n	Mean NIHSS	Mean Hgb level (g/dL)	Mean age (years)
Subcortical	22	3.7	12.3	67.5
Cortical/subcortical	8	8	13	68.6
Brainstem	6	6	12.3	70
Cortical	6	2.1	14	77.3
Cerebellar/brainstem	1	6	15.3	58
Cerebellar/subcortical	1	5	14.9	54

Analyzing the effects of gender, showed lower mean Hgb levels in the female group—which is an expected gender difference—yet the two groups did not show statistical differences in terms of the NIHSS scores (Table 6).

**Table 6:** Effect of gender on primary stroke outcome.

Gender	Mean age (SD) years	Mean NIHSS (SD)	Mean HgB (SD) (g/dL)
Females (*n* = 16)	67.06 (14.23)	5.8 (5.59)	11.45 (1.56)
Males (*n* = 28)	69.82 (13.14)	3.96 (5.59)	13.52 (1.87)
*p*-value	0.26	0.12	0.0001

## Discussion

The results of this retrospective study of a small cohort of patients diagnosed with AIS indicated that higher Hgb levels at the time of hospitalization were associated with greater stroke severity, as measured by the NIHSS score. No significant difference was observed between low and high Hgb levels in terms of discharge status (cured, improved, or deceased) or stroke type. However, a significant difference in the mean Hgb level was observed across the different classes of ischemic stroke. Additionally, the presence of atrial fibrillation significantly affected the severity of ischemic stroke.

### Hemoglobin level, stroke severity, and outcomes after acute ischemic stroke

In this study, higher Hgb levels at the time of hospital admission were associated with higher NIHSS scores, indicating greater stroke severity in patients with AIS. A few studies have shown more severe impairment in patients with elevated Hgb levels upon admission compared to those with normal Hgb levels after AIS. Furlan et al. retrospectively studied the data of 9,230 patients with AIS. They reported that a greater degree of neurological impairment (a Canadian Neurological Scale score of <8) was associated with higher Hgb levels on admission. Additionally, higher Hgb levels on admission were linked to a greater degree of disability and increased 7-day, 30-day, and 90-day mortality following AIS. However, low Hgb levels on admission were not significantly associated with impairment, consistent with the study results (3, 7, 9). The Framingham study with a 34-year follow-up showed that individuals with high hematocrit levels had an increased risk of morbidity and mortality due to cardiovascular diseases.

Moreover, a U-shaped relationship between hematocrit and the risk of stroke in older women demonstrated an increased risk of stroke at the extremes of Hgb levels, although no such association was observed among men (18, 19). Similar results were observed in other studies, such as the one conducted by Panwar et al., who reported an independent U-shaped relationship between low and high Hgb concentrations and the risk of stroke in women but not men (20). Barlas et al. found a U-shaped association between both low and high Hgb levels and short-term mortality in men with ischemic stroke (21). Gottesman et al. reported that patients with high Hgb levels on admission often developed neglect after a right-hemispheric ischemic stroke. They observed low and high Hgb levels might indicate worse outcomes after a stroke (22). A study of 1,213 patients with polycythemia vera with high Hgb levels found associations with a higher risk of stroke (measured by NIHSS) and worse stroke symptoms due to hemoconcentration and dehydration. High Hgb may be a marker of poor volume status (23).

In contrast to the abovementioned studies, most other studies have demonstrated an association between anemia and higher mortality after AIS.

Kellert et al. studied the data of 217 patients with AIS. They reported that patients with anemia on admission (defined as Hgb < 12 g/dL in women and < 13 g/dL in men) was approximately twice as frequent in patients with a poor outcome (defined as a modified Rankin Scale (mRS) score ≥ 3) compared to those with a favorable outcome (mRS ≤2) within the first 5 days of hospital stay. Patients with higher Hgb levels did not significantly differ regarding poor outcomes. In addition, anemic patients presented with more severe strokes, as reflected by a higher median NIHSS score (24). Tanne et al. (4) prospectively studied a cohort of 859 patients with ischemic or intracerebral hemorrhage. They reported that patients with anemia (defined as Hgb <13 g/dL in men and < 12 g/dL in women) exhibited the highest rates of poor outcomes at both 30 days and 1 year, while those in the middle quartiles of Hgb levels exhibited the lowest rates. Similar relationships were observed across sex (men versus women), age (younger versus older patients), and stroke types (ischemic stroke versus intracerebral hemorrhage), with no evidence of interaction effects. Additionally, anemia on admission was associated with increased mortality at 30 days and 1 year (4). Barlas et al. demonstrated that in women with ischemic stroke, low Hgb levels were significantly associated with mortality at five time points: inpatient, 1 month, 3 months, 6 months, and 1 year (21).

### Hemoglobin level and the TOAST stroke subtypes

This study found that according to the TOAST classification of ischemic stroke subtypes, small-vessel occlusion was the most common (47.7%), followed by large artery atherosclerosis 34%, cardio-aortic embolism 13.6%, and stroke of other etiologies (4.5%). These percentages differ from those reported in other studies: Kimberly et al. studied 259 patients with an ischemic stroke and found cardio-aortic embolism to be the most common subtype (42.9%), followed by large artery atherosclerosis (27.4%), undetermined causes (15.5%), other causes (11.8%), and small artery occlusion (2.2%). The TOAST stroke subtypes in their study demonstrated a statistically significant association with initial infarct volume (25). This study found no significant difference between Hgb levels (low versus high) among stroke types (cortical, subcortical, infratentorial, and subcortical). Similarly, Tanne et al. (4) reported no association between Hgb levels and stroke type (4).

### Effect of various comorbidities on the severity of ischemic stroke

This study included multiple variables in a linear regression model to assess their effects on the dependent variable (NIHSS score). These variables included diabetes, coronary artery disease, atrial fibrillation, hypertension, dyslipidemia, and a history of stroke. Most of these variables showed no significant effect on the severity of AIS. However, atrial fibrillation was the only comorbidity with a statistically significant effect (*p* < 0.05) on the severity of ischemic stroke.

Fischer et al. prospectively collected data from 266 patients and found that comorbidities had a significant impact on stroke outcomes. In addition to stroke severity, atrial fibrillation, coronary artery disease, and diabetes were identified as predictors of unfavorable outcomes after stroke (26).

### Study limitations

This study had several limitations that should be highlighted and acknowledged. First, the sample size of the study is small, making it difficult to detect subtle yet clinically relevant associations. This highlights the need for further larger, multi-center studies to confirm our findings. Second, potential confounding factors such as inflammatory markers, comorbidities, hydration status may have influenced hemoglobin levels and stroke severity outcomes but were not extensively controlled for this study. Third, the cross-sectional design of this study prevents causal inferences. However, a significant difference in mean hemoglobin levels was observed among TOAST stroke subtypes. Future large prospective studies with a more comprehensive assessment of stroke pathophysiology and hemoglobin variations over time are warranted to better elucidate the clinical implications of these findings.

Conclusion: The results of this study revealed a weak inverse correlation between Hgb levels and stroke severity, as determined by the NIHSS score. However, this correlation was not statistically significant. There was a remarkable difference in mean Hgb levels was observed across the TOAST stroke categories, with the atherosclerotic subtype having the highest levels, suggesting a potential link to stroke classification. Further studies with larger sample sizes are recommended to validate these findings.

## Data Availability

The raw data supporting the conclusions of this article will be made available by the authors, without undue reservation.
